# Cost-effectiveness of activated protein C in real-life clinical practice

**DOI:** 10.1186/cc6116

**Published:** 2007-09-06

**Authors:** Jean-François Dhainaut, Stéphanie Payet, Benoit Vallet, Lionel Riou França, Djillali Annane, Pierre-Edouard Bollaert, Yves Le Tulzo, Isabelle Runge, Yannick Malledant, Bertrand Guidet, Katell Le Lay, Robert Launois, the PREMISS Study Group

**Affiliations:** 1 Department of Intensive Care, Cochin Port-Royal University Hospital, AP-HP, René Descartes University, Paris 5, Paris, France; 2 REES France, Réseau d'Evaluation en Economie de la Santé, Paris, France; 3 Department of Anesthesiology and Intensive Care, University Hospital of Lille, University of Lille 2, Lille, France; 4 Department of Intensive Care, Raymond Poincaré Hospital, AP-HP, University of Versailles Saint-Quentin-en-Yvelines, Garches, France; 5 Department of Intensive Care, Central Hospital, University of Nancy, Nancy, France; 6 Department of Infectious Diseases and Medical Intensive Care, University Hospital of Rennes, Rennes, France; 7 Department of Intensive Care, La Source Hospital, Orléans, France; 8 Department of Anesthesiology and Intensive Care, University Hospital of Rennes, Rennes, France; 9 Department of Intensive Care, Saint Antoine Hospital, AP-HP, Pierre et Marie Curie University, Paris 6, Paris, France; 10 Members of the Protocole en Réanimation d'Evaluation Médico-économique d'une Innovation dans le Sepsis Sévère (PREMISS) study are listed in Appendix 1

## Abstract

**Background:**

Recombinant human activated protein C (rhAPC) has been reported to be cost-effective in severely ill septic patients in studies using data from a pivotal randomized trial. We evaluated the cost-effectiveness of rhAPC in patients with severe sepsis and multiple organ failure in real-life intensive care practice.

**Methods:**

We conducted a prospective observational study involving adult patients recruited before and after licensure of rhAPC in France. Inclusion criteria were applied according to the label approved in Europe. The expected recruitment bias was controlled by building a sample of patients matched for propensity score. Complete hospitalization costs were quantified using a regression equation involving intensive care units variables. rhAPC acquisition costs were added, assuming that all costs associated with rhAPC were already included in the equation. Cost comparisons were conducted using the nonparametric bootstrap method. Cost-effectiveness quadrants and acceptability curves were used to assess uncertainty of the cost-effectiveness ratio.

**Results:**

In the initial cohort (*n *= 1096), post-license patients were younger, had less co-morbid conditions and had failure of more organs than did pre-license patients (for all: *P *< 0.0001). In the matched sample (*n *= 840) the mean age was 62.4 ± 14.9 years, Simplified Acute Physiology Score II was 56.7 ± 18.5, and the number of organ failures was 3.20 ± 0.83. When rhAPC was used, 28-day mortality tended to be reduced (34.1% post-license versus 37.4% pre-license, *P *= 0.34), bleeding events were more frequent (21.7% versus 13.6%, *P *= 0.002) and hospital costs were higher (€47,870 versus €36,717, *P *< 0.05). The incremental cost-effectiveness ratios gained were as follows: €20,278 per life-year gained and €33,797 per quality-adjusted life-year gained. There was a 74.5% probability that rhAPC would be cost-effective if there were willingness to pay €50,000 per life-year gained. The probability was 64.3% if there were willingness to pay €50,000 per quality-adjusted life-year gained.

**Conclusion:**

This study, conducted in matched patient populations, demonstrated that in real-life clinical practice the probability that rhAPC will be cost-effective if one is willing to pay €50,000 per life-year gained is 74.5%.

## Introduction

Severe sepsis with multiple organ failure is a life-threatening systemic response to infection, leading to death in 34% to 65% of patients [1-5]. It is common in patients requiring intensive care in France, where more than 10% of admitted patients are affected [4]. Several studies have shown that high incidence of severe sepsis with attendant high mortality rates are associated with substantial health care costs [1,5].

Recombinant human activated protein C (rhAPC), drotrecogin alfa (activated), is a new treatment for severe sepsis. Evidence for the efficacy of rhAPC comes primarily from the pivotal PROWESS (Recombinant Human Activated Protein C Worldwide Evaluation in Severe Sepsis) study [6], a large, randomized, placebo-controlled trial. This study demonstrated a statistically significant, absolute reduction of 6.5% in 28-day mortality. *A priori *subgroup analyses showed that the relative risk for death progressively decreased with increasing number of organ failures [7]. Absolute reduction in mortality was higher in patients who had two or more organ failures (7.7%) than in the whole PROWESS population. Drotrecogin alfa (activated) has been licensed in the European Union since 2002 for the treatment of adult patients with severe sepsis and multiple organ failure, when added to best standard care.

However, the expenses linked to this new treatment have raised concerns about its cost-effectiveness. The costs associated with rhAPC in patients with severe sepsis and multiple organ failure include not only the acquisition cost of the drug (€7,500 per 70 kg patient for the full recommended 96-hour course) but also potential costs associated with bleeding episodes, hospitalization costs and (where deemed appropriate) long-term health care costs for additional survivors of severe sepsis. Such additional costs vary markedly in the published literature [8-14] as a result of country-specific factors as well as choice of modeling approach to estimate these costs. For instance, the resource utilization perimeter used to calculate the cost per patient who is treated or not treated with rhAPC can influence the estimate. However, in most of these models the cost of the intervention always remains at a level that would be regarded as cost-effective by most decision makers, especially in patients with an Acute Physiology and Chronic Health Evaluation (APACHE) II score exceeding 24 [8,9,11] or those with multiple organ failure [13,14].

Moreover, all cost-effectiveness studies of rhAPC used efficacy data extracted from the PROWESS trial, which probably do not reflect real-life practice at bedside [15]. In our study, PREMISS (Protocole en Réanimation d'Evaluation Médico-économique d'une Innovation dans le Sepsis Sévère), we aimed to determine whether the cost-effectiveness indicated by the PROWESS data could be replicated in real-life clinical practice. We prospectively observed patients' outcomes and actual hospital costs before and after rhAPC became available in France, and we established the real-life cost-effectiveness of rhAPC in patients with severe sepsis and multiple organ failure.

## Materials and methods

### Study design and patients

The primary objective of this national, prospective, observational study was to estimate the costs of treating patients with rhAPC and to compare these with the costs of treating patients without using rhAPC. The secondary objective was to determine the cost-effectiveness of rhAPC in real-life clinical practice. In the present study, effectiveness was estimated for the purposes of economical analyses only [16]; the efficacy of rhAPC has already been demonstrated in the PROWESS study [6]. No randomization was conducted so that none of the patients included after the treatment was made available on the French market suffered a loss of opportunity. In addition, because the costs were to be estimated in patients to whom rhAPC was prescribed in a real-life management setting, it was essential that the study interfered as little as possible with intensive care physicians' practices [17]. External validity (the ability of a study to yield results that are reproducible in other studies) was given preference over internal validity (the ability of a study to provide results that truly reflect the variables measured). Therefore, rather than reproducing the results of PROWESS, we aimed in the present study to ensure that its results could be generalized to routine intensive care practice throughout France.

A pre-post design was considered to be the most appropriate. Patients were included before (pre-license study phase) and after (post-license study phase) rhAPC had been made available in France (January 2003). Inclusion/exclusion criteria were defined in accordance with the rhAPC (Xigris^®^) label approved in the European Union. Eli-Lilly Company, Indianapolis, Indiana, USA. Collected data included demographic factors; clinical information and use of resources on admission, at enrolment and during the hospital course; and outcome at 28 days.

Based on estimated average costs of €31,800 and €39,500, respectively, in the pre-license and post-license phases (according to a French pharmaco-economic model [18]) and assuming a normal distribution of the costs, accrual of 340 patients was required in each study phase to detect a difference of €7,700 in the average costs with a first-degree risk α of 0.05 and a power β of 0.80. If the study objective had been to detect a difference of effectiveness (mortality), then we estimate from the PROWESS results that 600 patients per phase would have been required.

The two French Intensive Care Societies launched the study in 2002, at the request of the Health Ministry. Because the study did not influence the practices of the intensive care physicians, approval of an ethics committee was not required.

### Measurement of and reduction in recruitment bias

Given the absence of randomization, there is no guarantee that patients in the two study phases are comparable. We described the presence of recruitment bias by calculating the standardized differences in each baseline variable between the two groups [19]. In order to achieve an unbiased comparison of costs, we controlled for recruitment bias using the propensity score method [20,21]. The propensity score summarizes all observed baseline variables in a single figure. We then used the propensity score to construct a sample of comparable patients in the two phases using a matching process, the SAS^© ^'match' macro [22], to obtain an optimal match. More details of the propensity score approach are given in Appendix 2.

### Estimation and comparison of costs

Cost analyses were conducted from the point of view of the health care provider because treatment of patients with severe sepsis is almost exclusively dispensed by hospital services. Complete hospitalization costs were estimated from the College of Intensive Care Database Users (CUB-Rea) database [23] and from a multiple regression equation derived from a micro-costing study, based on 211 stays in intensive care unit (ICU) in 1996 in France [24]. The French information system used for medico-economic description and measurement of hospital activity (Programme de Médicalisation des Systèmes d'Information], which is based on medical unit summaries (Résumés d'Unité Médicale), provided the following data: age, sex, length of stay, diagnoses on admission and at discharge, and diagnostic/therapeutic procedures performed. The CUB-Rea database provided the following specific intensive care indicators: Simplified Acute Physiology Score (SAPS) II score, Omega score, McCabe score and admission type. Hospitalization costs considered in the micro-costing study included ICU costs and post-intensive care costs. The ICU costs can be subdivided into variable direct costs, such as tests (laboratory and imaging), small materials, drugs and blood products, and time spent by care staff (state registered nurse and health care assistant); fixed direct costs, such as time spent by medical nursing staff (calculated on a *pro rata *basis for the length of stay); and variable indirect costs such as restaurant services, laundry, pharmacy and administration. Post-intensive care costs are based on number of days, valued using the departmental tariff category.

The equation obtained [14] had a good determination coefficient (*R*^2 ^= 93%) and was expressed as follows:

CC = β_0 _+ (β_1 _× LOS) + (β_2 _× LOS × 1_DCR = 1_) + (β_3 _× Ω_TOT_) + (β_4 _× [SAPS2]^2^) + (β_5 _× 1_DCR = 1_)

Where CC is the total complete cost of the hospital stay (in 1996 French Francs), LOS is the length of stay in the ICU, Ω_TOT _is the total Omega score, SAPS2 is the SAPS II score, 1_DCR=1 _is the variable indicating death during intensive care, β_0 _is -8,881.50, β_1 _is 5,465.60, β_2 _is 3,715.10, β_3 _is 183.75, β_4 _is 5.27 and β_5 _is -18,078.50.

The way in which the equation was formulated implies that, for a short length of stay (<5 days), the cost incurred by survivors was greater than that generated by patients who die in intensive care. Beyond that given threshold, patients who eventually died in intensive care incurred increasing costs as their length of stay increased.

This general equation applied both to patients suffering from severe sepsis and to those suffering from other diseases, but it did not take into account the medical costs associated with administration of rhAPC. The acquisition costs of rhAPC were therefore added to the complete hospitalization costs, assuming that all of the connate costs associated with rhAPC administration (adverse events, longer term follow up and so on) were incorporated into the equation through the Omega score, the SAPS II score and the length of stay in intensive care. This was an essential assumption because it ensured that the total cost of patients receiving care with rhAPC was not underestimated. It was also a realistic assumption, because these three indicators were designed to represent activity in intensive care.

The year 2004 was chosen to harmonize all of the costs that have been calculated in this study because the most recent data available are for those patients admitted during that this year. The CUB-Rea equation was initially expressed in 1996 French Francs and inflation rates from the Institut National de la Statistique et des Etudes Economiques (INSEE) [25] were used to obtain nominal values for 2002, 2003 and 2004. All costs were then discounted for the year 2004, using a discount rate of 3.5%.

Cost comparisons were performed using the nonparametric bootstrap method [26], because cost variables are often asymmetric. A total of 10,000 samples of size *n *(starting sample size) obtained from the empirical distribution function of costs was generated by drawing, with replacement, *n *individuals randomly from the initial sample. The mean costs in each bootstrap sample were calculated for both groups, together with the difference between the two mean costs. We then tested whether this difference was significantly different from 0.

### Estimation of effectiveness

The effectiveness metric was life expectancy at 28 days after onset of sepsis. However, this data point was not directly available because only mortality at 28 days was recorded in the case report forms. The life expectancy of survivors was therefore estimated using the McCabe score. A set of assumptions was made [14]. First, patients suffering from a short-term fatal disease (1 year) were allocated a life expectancy of 0.5 years. Second, the life expectancy of patients suffering from a long-term fatal disease (5 years) was estimated to be 3 years. Third, the life expectancy of patients without fatal co-morbidities was calculated from the life expectancy of the French general population published in the INSEE tables [27], grouped by age and sex for the year 2003. One study [28] estimated that the life expectancy of patients who had suffered severe sepsis was reduced by half as compared with people of the same age and sex in the general population. The life expectancy extracted from the INSEE tables was therefore divided by 2 for this patient category.

Life expectancy was then adjusted with respect to quality of life to obtain a quality-adjusted life-year (QALY) gained outcome. Studies evaluating quality of life after intensive care stay reported a range of coefficients from 0.6 to above 0.8 [8,9,29,30]. The lowest coefficient (0.6) was used in the present study.

Although most analysts agree that costs should be discounted in any study that is conducted over a period of longer than 1 year, there is no consensus on whether the consequences or benefits of intervention should be discounted and at what rate. It was therefore decided not to discount the measure of effectiveness.

### Cost-effectiveness ratio

Unlike the previous rhAPC cost-effectiveness estimations, our cost-effectiveness ratio is derived from a trial collecting both effectiveness and cost data, and not from a model combining different data sources. The approach taken to deal with uncertainty in the estimates is consequently statistical and not based on sensitivity analyses.

The difficulty in obtaining the distribution of a ratio has been discussed elsewhere in the literature [31]. We used once again the nonparametric bootstrap method, by generating 10,000 bootstrap samples of the mean effectiveness, the mean cost and the cost-effectiveness ratio. The results were represented in a cost-effectiveness plane, linking effectiveness to costs.

From the same bootstrap samples, an acceptability curve of rhAPC was also constructed. This curve shows the probability that the treatment is efficient according to the decision makers' willingness to pay. For a willingness to pay of λ, this probability is equal to the proportion of bootstrap samples in which the ratio calculated is less than λ. This curve provides another measure of uncertainty that is linked to the overview estimate of the cost-effectiveness ratio [32].

## Results

### Patient characteristics in the initial cohort (1,096 patients)

Overall, 85 participating ICUs recruited 1,096 patients with severe sepsis and multiple organ failure. The inclusion rate during the post-license phase when rhAPC came into use was much lower than during the pre-license phase: 509 patients were enrolled between July 2002 and December 2002 (before the French license had been obtained), and 587 patients between January 2003 and December 2004 (after the French license had been obtained). The patients' baseline characteristics are provided in Table 1, overall and by study phase. The overall cohort characteristics corresponded to those of the population targeted in the European recommendations for using rhAPC. Patients were severely ill and were at high risk for death, and had failure of two or more organs. The mean SAPS II score [33] was 56.6 ± 18.6, which corresponds to a predicted hospital mortality of 61%, and the mean Logistic Organ Dysfunction score [34] was 7.67 ± 2.82. Neurological failure was excluded from the calculation of organ failure because most of the patients were sedated at enrolment in both phases. Despite this, the observed mean number of organ failures in the initial cohort was greater than 3 (3.21 ± 0.86).

**Table 1:** Patient characteristics in the initial cohort

Characteristic	All patients (*n *= 1,096)	Pre-license (*n *= 509)	Post-license (*n *= 587)	*P*
Demographics				
Age (yrs)	60.8 ± 16.3	63.9 ± 15.1	58.1 ± 16.8	<0.0001
> 60 yrs	57.9	64.1	52.5	0.0001
Male	62.0	61.5	62.5	0.7265
Weight (kg)	73.9 ± 17.4	73.5 ± 17.3	74.2 ± 17.4	0.5546
Prior location				0.0702
Medical or surgical department	40.4	44.0	37.3	
Emergency department	28.4	27.1	29.5	
Another acute care hospital	22.6	19.8	25.0	
Home	8.6	9.1	8.2	
Reason for ICU admission				0.9168
Medical	71.7	72.1	71.4	
Surgical	27.0	26.5	27.4	
Trauma	1.3	1.4	1.2	
Disease severity				
SAPS II on admission	56.6 ± 18.6	56.9 ± 19.1	56.2 ± 18.1	0.5427
LOD score at enrolment^a^	7.67 ± 2.82	7.44 ± 2.93	7.87 ± 2.71	0.0112
Organ failure at enrolment^a^	3.21 ± 0.86	3.10 ± 0.86	3.31 ± 0.85	<0.0001
Acute lung injury	2.1 ± 1.1	1.9 ± 1.2	2.2 ± 1.0	<0.0001
Acute renal failure	3.4 ± 1.7	3.3 ± 1.8	3.4 ± 1.7	0.3777
Coagulopathy	0.3 ± 0.7	0.3 ± 0.6	0.3 ± 0.7	0.0629
Acute liver failure	0.3 ± 0.5	0.3 ± 0.5	0.3 ± 0.5	0.1831
Acute cardiovascular Failure	1.6 ± 1.3	1.7 ± 1.3	1.6 ± 1.2	0.4399
Shock at enrolment	93.7	92.5	94.7	0.1375
Co-morbid conditions				
McCabe				<0.0001
0	36.4	30.8	41.5	
1	35.9	34.2	37.5	
2	21.5	26.0	17.5	
3	6.1	9.0	3.6	
Chronic renal failure	6.3	7.7	5.0	0.0649
Chronic liver disease	4.2	4.4	4.1	0.8593
Congestive cardiomyopathy	13.0	14.1	12.0	0.3018
COPD	14.3	14.7	14.1	0.7824
Diabetes mellitus	6.5	6.8	6.2	0.7123
Immunosuppressive treatment	6.1	5.7	6.4	0.6653
Chemotherapy	3.2	3.9	2.6	0.1982
Metastatic cancer	5.0	6.4	3.8	0.0553
Haematological malignancies	3.3	4.2	2.6	0.1450
HIV	1.8	1.4	2.1	0.4018
Infection site				
Lung	49.2	50.1	48.4	0.5867
Intra-abdominal	26.2	27.6	25.0	0.3454
Urinary tract	9.8	12.0	7.9	0.0273
CNS	4.9	2.7	6.7	0.0032

Values are expressed mean ± standard deviation or proportions of patients. ^a^Neurological failure excluded. CNS, central nervous system; COPD, chronic obstructive pulmonary disease; ICU, intensive care unit; LOD, Logistic Organ Dysfunction; SAPS, Simplified Acute Physiology Score.

### Presence and correction of recruitment bias

Of the 81 standardized differences calculated, 43 exceeded the 10% threshold, reflecting an imbalance between the two phases. Even though the patients recruited in the two phases had similar severity indices (SAPS II and Logistic Organ Dysfunction scores), they did not have the same degree of severity. More patients in the post-license group had respiratory failure, whereas patients in the pre-license group had more severe neurological disorders. In addition, patients recruited for rhAPC treatment were younger and less likely to die within the year. More patients in the pre-license phase were admitted through internal transfer into the ICU. Also, more of them were suffering from endocardiovascular and urinary tract infections.

**Figure 1:**
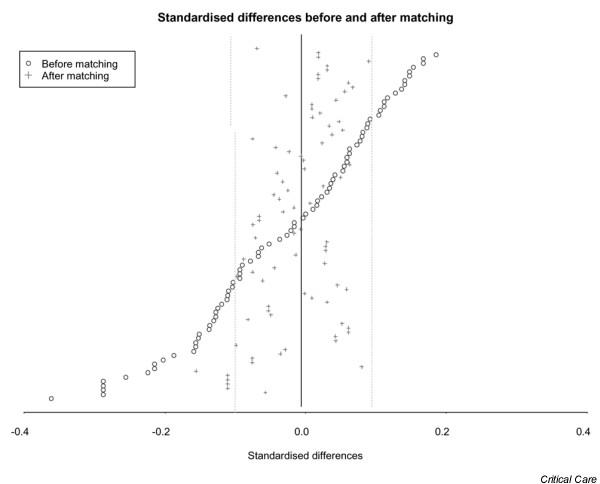
Changes in standardized differences before and after matching.

Matching by use of the propensity scores produced a sample of 840 patients (420 in each phase). The new sample corresponded to 76.6% of the initial cohort. The patients' characteristics are presented in Table 2. Overall, the mean age was 62.4 ± 14.9 years, the mean SAPS II score was 56.7 ± 18.5, and mean number of organ failures was 3.20 ± 0.83. Recruitment biases were markedly reduced or nearly absent, because only five variables (among 81) still exhibited a standardized difference exceeding 10% (Figure 1). These variables reflected that patients aged 80 years or older (difference 14.9%) and nonventilated patients (difference 10.5%) were more numerous in the pre-license phase. Subsequent analyses were conducted in this matched population.

**Table 2:** Patient characteristics in the matched sample

Characteristic	All patients (*n *= 840)	Pre-license (*n *= 420)	Post-license (*n *= 420)	*P*
Demographics				
Age (yrs)	62.4 ± 14.9	62.7 ± 15.3	62.0 ± 14.4	0.4584
> 60 yrs	61.5	61.4	61.7	0.9435
Male	62.4	60.7	64.1	0.3187
Weight (kg)	74.6 ± 17.4	74.1 ± 17.6	75.1 ± 17.1	0.4192
Prior location				0.8676
Medical or surgical department	40.9	41.9	40.0	
Emergency department	28.7	27.4	30.0	
Another acute care hospital	21.2	21.4	21.0	
Home	9.2	9.3	9.0	
Reason for ICU admission				0.8428
Medical	69.9	70.7	69.0	
Surgical	29.0	28.3	29.8	
Trauma	1.1	1.0	1.2	
Disease severity				
SAPS II on admission	56.7 ± 18.5	56.8 ± 19.1	56.6 ± 18.0	0.8833
LOD score at enrolment^a^	7.60 ± 2.82	7.51 ± 2.91	7.70 ± 2.73	0.3384
Organ failure at enrolment^a^	3.20 ± 0.83	3.15 ± 0.84	3.25 ± 0.82	0.0676
Acute lung injury	2.1 ± 1.1	2.1 ± 1.1	2.2 ± 1.1	0.1922
Acute renal failure	3.3 ± 1.7	3.3 ± 1.8	3.4 ± 1.7	0.5274
Coagulopathy	0.3 ± 0.6	0.2 ± 0.6	0.3 ± 0.6	0.7368
Acute liver failure	0.3 ± 0.5	0.3 ± 0.5	0.3 ± 0.5	0.5669
Acute cardiovascular Failure	1.6 ± 1.3	1.6 ± 1.3	1.6 ± 1.3	0.6664
Shock at enrolment	94.3	93.3	95.2	0.2344
Comorbid conditions				
McCabe				0.4541
0	35.1	34.8	35.4	
1	36.6	34.6	38.7	
2	22.7	24.2	21.2	
3	5.6	6.4	4.7	
Chronic renal failure	5.9	6.5	5.3	0.4775
Chronic liver disease	3.5	3.6	3.4	0.8552
Congestive cardiomyopathy	14.0	14.5	13.5	0.6684
COPD	15.4	14.7	16.1	0.5743
Diabetes mellitus	6.5	6.3	6.7	0.7854
Immunosuppressive treatment	4.8	4.8	4.8	0.9938
Chemotherapy	2.8	3.1	2.4	0.5259
Metastatic cancer	5.6	6.3	4.9	0.3761
Haematological malignancies	2.5	2.6	2.4	0.8208
HIV	5.9	6.5	5.3	0.3511
Infection site				
Lung	51.8	51.1	52.5	0.7078
Intra-abdominal	27.1	27.1	27.1	0.9987
Urinary tract	10.1	11.1	9.1	0.3417
CNS	3.3	3.0	3.5	0.7432

Values are expressed mean ± standard deviation or proportions of patients. ^a^Neurological failure excluded. CNS, central nervous system; COPD, chronic obstructive pulmonary disease; ICU, intensive care unit; LOD, Logistic Organ Dysfunction; SAPS, Simplified Acute Physiology Score.

### Hospital course, burden of care and costs

Table 3 summarizes hospital course, burden of care and costs in the matched population. Patients in the post-license phase stayed longer in the ICU (24.4 days versus 21.3 days, *P *= 0.002) and tended to stay longer in hospital (40.4 days versus 37.9 days, *P *= 0.09) than did those in the pre-license phase. The burden of care was higher in the post-license phase, as assessed using the relative cost index (2,862 versus 2,430, *P *< 0.05) and the Omega score (427 versus 373, *P *< 0.05). A multivariate model showed that the increase in burden of care (measured by relative cost indices) was essentially due to the increase in length of stay in the ICU (*P *< 0.0001). However, after adjustment on the length of stay in the ICU, the difference between both study phases in the burden of care remained statistically significant (*P *= 0.048). Similar results were found when the burden of care was measured using the Omega score. The burden of care during the post-license phase when using rhAPC was therefore higher, due to both length of stay in the ICU and daily resource utilization.

**Table 3:** Burden of care and hospitalization costs in the matched patients

	All patients (*n *= 840)			Survivors (*n *= 471)			Nonsurvivors (*n *= 369)		
	
	Pre-license	Post-license	Pre-Post license difference (95% CI)	Pre-license	Post-license	Pre-Post license difference (95% CI)	Pre-license	Post-license	Pre-Post license difference (95% CI)
Omega score	373	427*	54 (9.12 to 98.03)	380	433	53 (-10 to 112)	364	418	54 (-13 to 121)
Reference cost index	2,430	2,862*	432 (187 to 662)	2,254	2,667*^†^	413 (96 to 722)	2,648	3,121*^†^	473 (9 to 936)
ICU stay (day)	21.3	24.4*	3.1 (0.32 to 5.92)	23.8	26.7^†^	2.9 (-0.90 to 6.57)	18.2	21.3^†^	3.1 (-0.89 to 7.25)
Hospital stay (day)	37.9	40.4	2.5 (-1.79 to 6.84)	49.2	51.1^†^	1.9 (-4.41 to 8.37)	24.6	27.5^†^	2.9 (-2.02 to 7.95)
Costs -rhAPC (€)	36,717	41,144	4,427 (-85 to 8,991)	35,575	39,172	3,597 (-1,737 to 8,680)	38,095	43,729	5,634 (-2,005 to 13,380)
Total costs (€)	36,717	47,870*	11,153 (6,601 to 15,709)	35,575	46,752*	11,177 (5,863 to 16,313)	38,095	49,336*	11,241 (3,433 to 19,084)

Values are expressed means and 95% confidence interval (CI) on the means. *P < 0.05 pre-license versus post-license. ^†^*P *< 0.05 post-license survivors versus nonsurvivors. ICU, intensive care unit; rhAPC, recombinant human activated protein C; -rhAPC, without rhAPC acquisition costs.

The increase in drug costs observed in the post-license phase was related not only to the acquisition of rhAPC itself (€6,717 on average) but also to that of other therapies, including antimicrobial agents (€1,900 versus €1,321, *P *< 0.05). Blood and plasma transfusion costs were also higher in the post-license phase (€1,043 versus €751, *P *< 0.05), the occurrence of transfusions being essentially due to the bleeding events observed (at least one event for 21.67% versus 13.57% of patients; *P *< 0.05). Overall, complete hospitalization costs were higher in the post-license phase (€47,870 versus €36,717, *P *< 0.05). Sixty per cent of this difference was attributable to the rhAPC acquisition costs.

When survivors and nonsurvivors in the post-license phase were compared (Table 3), the length of stay in ICU and hospital was lower in nonsurvivors (*P *< 0.05). However, the total hospitalization costs in the post-license phase, whether rhAPC acquisition costs were included or not, were similar in survivors and nonsurvivors.

### Survival

The two study phases did not differ significantly in 28-day mortality (34.1% post-license versus 37.4% pre-license, *P *= 0.34). The mean life expectancy was 6.68 ± 7.33 years for patients in the post-license phase and 6.13 ± 7.20 years for patients in the pre-license phase. This difference (0.55 years gained when rhAPC was used) was also not significant (*P *= 0.22). By applying a quality of life coefficient of 0.6, patients in the pre-license phase gained 3.68 ± 4.32 QALYs and those in the post-license phase gained 4.01 ± 4.40 QALYs, resulting in a difference of 0.33 QALYs gained when rhAPC was used.

### Cost-effectiveness estimates

**Figure 2:**
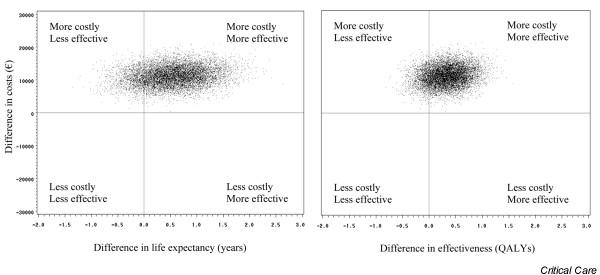
Cost-effectiveness of rhAPC. The figure shows the distribution of the incremental cost-effectiveness ratios in terms of life expectancy (left panel) and of quality-adjusted life-years (QALY; right panel) after 10,000 bootstrap replicates. Quadrants to the right of the y-axis represent the region where treatment with recombinant human activated protein C (rhAPC) is associated with a net gain in effect (85.92%). Quadrants above the x-axis represent the region where treatment is associated with a net increase in cost (100%). Both distributions were thus predominantly in the 'more costly, more effective' upper right quadrant.

**Figure 3:**
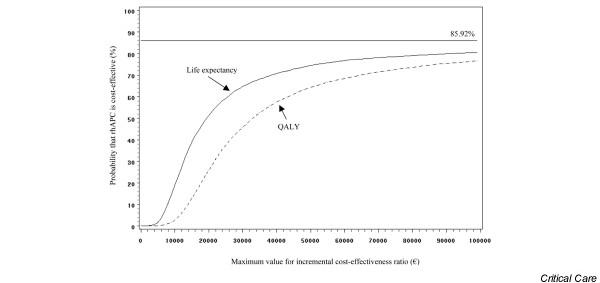
Cost-effectiveness acceptability curves of rhAPC. The curves represent the probability that treatment with recombinant human activated protein C (rhAPC) is associated with a cost per life-year gained and a cost per quality-adjusted life-years (QALY) gained that are lower than the corresponding incremental cost-effectiveness ratios shown on the x-axis. There was a 74.5% probability that the use of rhAPC would be cost-effective if there were willingness to pay €50,000 per life-year gained and a 64.3% probability if there were willingness to pay €50,000 per QALY gained.

Without adjusting for quality of life, incremental cost-effectiveness of rhAPC was €20,278 per life-year gained. After adjusting for quality of life, it was €33,797 per QALY. Figure 2 shows the distribution of incremental cost-effectiveness ratios in terms of life expectancy and of QALYs after 10,000 bootstrap replicates. Quadrants to the right of the y-axis represent the region where treatment with rhAPC is associated with a net gain in effect (85.92%). Quadrants above the x-axis represent the region where treatment is associated with a net increase in cost (100%). Both distributions were thus predominantly in the 'more costly, more effective' upper right quadrant. The acceptability curves (Figure 3) show, for each willingness to pay, the probability that rhAPC would be acceptable (the probability that the ratio is below the willingness to pay). The asymptote of the acceptability curves was not equal to 1, simply because the bootstrap samples included data in which rhAPC added to best standard care was less effective than best standard care alone. The asymptote was equal to the proportion of bootstrap samples for which the number of (quality-adjusted) life-years gained was greater in the post-license phase than in the pre-license phase (85.92%). There was a 74.5% probability that the use of rhAPC in septic patients with multiple organ failure would be cost-effective if there were willingness to pay €50,000 per life-year gained. The probability was 64.3% if there were willingness to pay €50,000 per QALY gained.

## Discussion

This study shows, for the first time in real-life clinical practice, that rhAPC is cost-effective in patients with severe sepsis and multiple organ failure. There was a 74.5% probability that rhAPC would be cost-effective if there were willingness to pay €50,000 per life-year gained. The results also suggest that ICU physicians preferentially targeted the most severely ill patients with reasonable life expectancy for rhAPC treatment.

### Target for rhAPC treatment in clinical practice and selection bias

ICU physicians enrolled patients using the same inclusion/exclusion criteria (defined according to the approved rhAPC label) throughout the study. However, patients in the post-license phase (that is, patients who received rhAPC) were younger and had fewer underlying diseases but more organ failures at study entry than those in the pre-license phase (initial cohort). We speculate that the physicians, when giving such an expensive drug carrying increased risk for bleeding, excluded the very elderly (>80 years), patients with advanced underlying disease (McCabe 3) and patients with fewer than three organ failures, in order to target treatment to the most severely ill patients with reasonable life expectancy if they survived the episode of severe sepsis. It is interesting to note that rhAPC was not over-used, even though two-thirds of the drug acquisition costs were met by the Ministry of Health throughout the study.

The markedly longer period of recruitment after the French license had been obtained (24 months versus 6 months for the pre-license phase) also advocates for increased selection of patients to receive rhAPC. Furthermore, although the occurrence of all bleeding events differed significantly between the two phases (13.6% versus 21.7%), it was still less than that observed in the patients with multiple organ failure in the PROWESS trial in both placebo and rhAPC groups (17.9 versus 25.4%) [7]. This could either be due to the fact that, in our observational study, adverse events were not reported as rigorously as in a trial setting or (more likely) to selection of patients with no serious risk for bleeding in real-life clinical practice.

It is also worth noting that the reduction in 28-day mortality in the post-license phase, when rhAPC was used, was modest despite the fact that a markedly larger proportion of patients were treated with low-dose steroids in the post-license phase than in the pre-license phase (80.5% versus 55.0%, *P *< 0.0001), probably linked to the higher severity of illness. Indeed, low doses of hydrocortisone and fludrocortisone have been shown to reduce significantly the risk for death in patients with septic shock and relative adrenal insufficiency, without increasing adverse events [35]. No interaction between steroids and rhAPC has been reported to our knowledge, and in the PROWESS trial mortality was lower with rhAPC than with placebo, whether steroids were given at baseline or during the infusion period, or were not given at all [36,37].

### Dealing with selection bias

Recruitment biases inherent to nonrandomized study designs are well recognized. Because we were aware, at the time when the study was designed, that imbalance in patient characteristics was likely to occur and of the resulting incomparability of the groups in terms of resource use and hence of costs in the initial cohort, we took preventative measures. I was our intention that use of the propensity score would control for these biases. The main limitation of the propensity score is that it can only take into account observed biases [20,21]. The case record forms were thus designed to allow recording of all initial clinical characteristics deemed likely to affect effectiveness, resource utilization and costs. Forty-six such variables were identified. The probability that a confounding factor was left out is therefore quite low. As a result, in the sample of patients matched with respect to propensity score, recruitment biases were markedly reduced or were almost entirely removed. No statistically significant differences between the two phases were found. Consequently, we are confident that the observed differences with regard to rhAPC cost-effectiveness were not related to the characteristics of the patients.

We believe selection bias is smaller in a pre-post design than in a post-license only study matching untreated patients to rhAPC treated patients, because rhAPC is not an option in the pre-license phase.

### Relation to other studies

The present study confirms the discrepancy that is often observed between rigorously planned clinical trials and real-life clinical practice. Cost-effectiveness of rhAPC in our study was less favourable than that described previously in the literature. However, and in contrast with our study, all other studies used the effectiveness data of the randomized, double-blind, placebo-controlled clinical trial PROWESS [6]. For comparison, the incremental cost-effectiveness ratio per life-year gained and per QALY gained were €20,278 and €33,797, respectively, in the present study. In the other studies, the ratio in the most severely ill patients (APACHE II score > 24 for North America, and multiple organ failure for Europe) was around US$15,000 in the North American studies [8-11] and €13,000 in the European studies [12-14] per life-year gained. The corresponding values per QALY gained were US$30,000 and €22,000, respectively.

The greater cost-effectiveness ratio obtained in the present study was due to a lower absolute reduction in the 28-day mortality between matched groups when compared with PROWESS (-3.3% versus -6.1% overall and -7.7% in the subgroup with multiple organ failure) [6,7] rather than to hospital costs. This was unexpected. Indeed, the very severely ill patients theoretically represented a population more likely than the PROWESS global population to benefit from rhAPC, because reduction in mortality was demonstrated to be the highest in patients with an APACHE II score greater than 24 [38] and those with multiple organ failure enrolled in PROWESS [7]. When compared with the global population [6] and the subgroup with multiple organ failure [7] of PROWESS, the 840 patients in the matched population of PREMISS had different baseline characteristics. They exhibited higher predicted mortality (61.3% in PREMISS versus 52.6% in PROWESS global and 55.9% in PROWESS multiple organ failure, calculated using the mean SAPS II or APACHE II score) and a higher number of organ failures (3.20 versus 2.40 and 2.92, respectively), although neurological failure was not taken into account in the present study. Also, our study population included a greater proportion of patients undergoing mechanically ventilation patients (94.6% versus 75.5% and 81.1%), a greater proportion of patients with shock (94.3% versus 71.0% and 82.4%) and a greater proportion of patients requiring vasopressor agents (88.6% versus 70.9% and 72.7%).

This discrepancy may be explained as follows. First, the effect of rhAPC on mortality might be limited in the most severely ill patients. However, this hypothesis would not be consistent with the PROWESS subgroup analyses [38], which showed that absolute reduction in 28-day mortality was lower in patients with failure of one or two organs (1.7% and 5.3%, respectively) than in patients with failure of three or four organs (8.2% and 7.9%, respectively). Second, the small recruitment bias that persisted after the matching process may be responsible for the apparent lower efficacy of the drug when compared with the findings in PROWESS. This is unlikely because the only variables concerned exhibited small standardized differences (below 15%) and should counterbalance each other; the very elderly (more numerous by 14.9% pre-license) are more vulnerable than the youngest, whereas nonventilated patients (more numerous by 10.1% pre-license) are less vulnerable than mechanically ventilated patients. Third, physicians might have delayed administration of rhAPC after sepsis onset in the face of a transient stabilization of the patient after conventional treatment. Indeed, the drug when administered after the first 24 hours of the onset of sepsis has been shown to have apparently lower efficacy [39,40]. However, 70% of the patients enrolled in the post-license phase received rhAPC within the first day of admission to the ICU.

A fourth reason for the discrepancy between the findings of PREMISS and those of PROWESS is that the decrease in mortality observed in PROWESS might have overestimated the real effect of the drug. This is because the proportions of patients who had septic shock, who were being treated with vasopressor agents, who were receiving mechanical ventilation, or who suffered from underlying diseases, were higher in the placebo group than in the rhAPC group [6]. Larger differences in baseline underlying diseases were observed in the placebo subgroup with multiple organ failure [7], in particular in liver and cardiovascular diseases, which are known to have a strong influence on mortality rates in patients with severe sepsis after the first 3 days [3,4]. Although no difference was statistically significant, these imbalances slightly favour the rhAPC group, especially in patients with multiple organ failure [36,41]. The findings of our study may therefore represent the real-life reduction in mortality resulting from rhAPC use.

The greater cost-effectiveness ratio observed compared with other studies might also be due to increased hospital costs, but to a limited extent only. In the matched population, rhAPC added to best standard care significantly increased resource use and total hospital costs in both survivors and nonsurvivors of severe sepsis with multiple organ failure. This was related to both greater length of ICU stay and more intense daily intensive care. Among the seven economic studies evaluating rhAPC, only that of Angus and coworkers [9] reported on hospital course and burden of care (assessed using the 28-item version of Therapeutic Intervention Scoring System). No differences between the placebo and treatment groups were observed for the length of ICU stay or the burden of care in the cost cohort (US patients of the PROWESS trial). The reason for these apparently conflicting results is unknown. We presume that the rhAPC-related improvement in status of our very severely ill patients required a longer ICU stay and greater intensity of daily intensive care than in the PROWESS trial. However, the incremental cost per patient treated was similar in both studies (US$9,800 versus €11,153) and was significantly different only when the acquisition cost of the drug was taken into account in the hospitalization costs.

### Limitations of the study

To summarize, the main limitations of the present study are as follows.

First, there was no randomization; this was in order to avoid denying patients an opportunity to receive a treatment that had been deemed effective in a previous trial [6]. Our study shows some evidence of selection bias, which we controlled using propensity score matching.

The second limitation is the choice of the control group. In a pre-post design, historical control individuals are used. Because the control patients were recruited only a few months before the first treated patients, and exploratory analyses did not show signs of temporal trends, we have no reason to believe that the results were biased by changes in practice over time.

Third, the sample size was tailored for cost comparisons. As a result, the study is underpowered to deal with effectiveness issues. The absence of a significant difference in effectiveness in the present study is no reason not to perform a cost-effectiveness analysis, although it adds to the variability in the cost-effectiveness estimate.

The final limitation is the absence of follow up of patients once they had left the hospital. Some assumptions had to be made regarding their expected life expectancy and quality of life. These assumptions are based on those made in previous cost-effectiveness models [14]. However, because the assumptions were the same for both treatment strategies, the final estimates are much less sensitive to a change in these parameters than to a change in 28-day mortality.

## Conclusion

This prospective, observational study shows that, in real-life clinical practice, rhAPC is cost-effective in the management of patients with severe sepsis with multiple organ failure. It is the first reported cost-effectiveness study of rhAPC that does not derive its primary data from one large pivotal study.

## Key messages

• Complete hospitalization costs were higher in the post-license phase (€47,870 versus €36,717); 60% of this difference was attributable to the rhAPC acquisition cost.

• There was a 74.5% probability that rhAPC would be cost-effective if there were willingness to pay €50,000 per life-year gained.

• Without adjusting for quality of life, the incremental cost-effectiveness of rhAPC was €20,300 per life-year gained; after adjusting for quality of life it was of €33,797 per QALY.

• The cost-effectiveness ratio is higher than the previously published PROWESS-based estimates. This is because of a lower absolute reduction in 28-day mortality (-3.3% in our study versus -6.1% overall and -7.7% in the subgroup with multiple organ failure in the PROWESS study) rather than being due to hospital costs.

• These less favourable estimates confirm the discrepancy between rigorously planned protocol trials and real-life clinical practice.

## Abbreviations

CUB-Rea = College of Intensive Care Database Users; ICU = intensive care unit; PREMISS = PROWESS = Recombinant Human Activated Protein C Worldwide Evaluation in Severe Sepsis; QALY = quality-adjusted life-year; rhAPC = recombinant human activated protein C; SAPS = Simplified Acute Physiology Score.

## Competing interests

J-FD has served as paid consultant for serving in an advisory board for GlaxoSmithKline, Lilly, and AstraZeneca, and for participating as a speaker in scientific meetings organized by GlaxoSmithKline and Lilly. BV has served as paid technical support for Edwards Life Sciences. All other authors declare that they have no competing interest.

## Authors' contributions

J-FD and BV obtained the funding. J-FD, BV, and RL conceived the study and participated in its design and coordination. BG participated in its design. KL developed a study-specific online data acquisition system and participated in the data management. SP and LRF carried out the statistical analysis. RL carried out the economical analysis. J-FD, RL, LRF and SP drafted the manuscript. All authors read and approved the final manuscript.

## Appendix I: the PREMISS Study Group

### Advisory board

The French Speaking Intensive Care Society (Société de Réanimation de Langue Française [SRLF]): C Brun-Buisson, B Guidet and J-F Dhainaut.

The French Society of Anaesthesia and Intensive Care (Société Française d'Anesthésie – Réanimation [SFAR]): A Lepape, C Martin and B Vallet.

Pharmaco-economic evaluation team: I Durand Zaleski and R Launois.

### Contributing centres

All of the contributing centres are in France: M Slama (Centre Hospitalier Universitaire [CHU], Amiens); P Asfar, A Kouatchet, L Beydon and JC Granry (CHU, Angers); JP Sollet and B Bleichner (CH, Argenteuil); JM Rodolfo, F Jaulin, L Mallet and D Raffier (CH, Auch); Y Cohen and M Samama (CHU Avicenne, Bobigny); A Boillot, G Capellier and JC Navellou (CHU, Besançon); C Gatecel (CH, Béziers); P Montravers and M Blaise (CHU Jean Verdier, Bondy); Y Castaing, O Pillet and G Gbikpi-Benissan (CHU Pellegrin Tripode, Bordeaux); L Holzapfel (CH, Bourg en Bresse); JM Boles and A Renault (CHU, Brest); C Daubin, P Charbonneau, JL Gérard and C Eustratiades (CHU, Caen); F Brivet, A Descorps-Declère and AS Dumenil (CHU Antoine-Béclère, Clamart); P Schoeffler, JE Bazin and B Souweine (CHU, Clermont-Ferrand); J Marty (CHU Beaujon, Clichy); D Dreyfuss and JD Ricard (CHU Louis Mourier, Colombes); C Brun-Buisson (CHU Henri Mondor, Créteil); P Sanjean (CH, Dax); B Blettery, JP Quenot, M Freysz and A Chomel (CHU, Dijon); M Kaidomar (CH, Frejus); D Annane and D Orlikowski (CHU, Garches); D Barnoud, C Jacquot and JF Payen (CHU, Grenoble); P Haglund and O Lesieur (CH, La Rochelle); D Thevenin and C Poisson (Centre Hospitalier Régional [CHR], Lens); A Durocher and F Saulnier (CHU Calmette, Lille); B Vallet and PA Rodie Talbere (CHU Huriez, Lille); F Fourrier and J Mangalaboyi (CHU Salengro, Lille); D Robert and I Mohammedi (CHU Edouard Herriot, Lyon); C Guérin, M Badet, JP Viale and P Branche (CHU Croix-Rousse, Lyon); JC Manelli and J Billot (CHU Conception, Marseille); C Martin and F Antonini (CHU Nord, Marseille); J Auffray (CHU Sainte Marguerite, Marseille); JF Poussel (CH Metz); PE Bollaert, A Cravoisy, PM Mertes, G Audibert and C Charpentier (CHU, Nancy); M Pinaud, R Champin and D Villers (CHU, Nantes); C Bengler, C Arich, C Gervais, JE Delacoussaye and JY Lefrant (CHU, Nîmes); G Bernardin, H Hyvernat, D Grimaud and C Ichai (CHU, Nice); T Boulain and I Runge (CHR, Orléans); D Benhamou, C Ract, J Duranteau, C Richard and JL Teboul (CHU Bicêtre, Paris); JM Desmonts, N Kermarrec, B Regnier and B Mourvillier (CHU Bichat, Paris); JF Dhainaut, N Marin and J Charpentier (CHU Cochin, Paris); JL Pourriat and H Dermine (CHU Hôtel-Dieu, Paris); D Payen and J Mateo (CHU Lariboisière, Paris); P Carli, V Mahe and H Nguyen (CHU Necker, Paris); C Gibert and CE Lyut (CHU Pitié-Salpêtrière, Paris); A Lienhart, JP Masini, J Pham and B Guidet (CHU Saint-Antoine, Paris); J Carlet, O Gattoliat and B Misset (Fondation Hôpital Saint Joseph, Paris); L Jacob, S Boudaoud, JR Legall and B Schlemmer (CHU Saint-Louis, Paris); JY Fagon (CHU Pompidou, Paris); F Bonnet and JP Fulgencio (CHU Tenon, Paris); G Janvier and C Fleureau (CHU Bordeaux, Pessac); A Lepape, PY Gueugniaud, J Bohé, H Thizy, D Jacques and G Fournier (CHU Lyon Sud, Pierre-Bénite); R Robert (CHU, Poitiers); S Lavoué, Y Le Tulzo, Y Malledant, A Maurice and P Seguin (CHU, Rennes); G Bonmarchand, K Clabault, J.C Chakarian and B Veber (CHU, Rouen); C Auboyer and R Jospe (CHU Nord, Saint-Etienne); F Zeni (CHU Bellevue, Saint-Etienne); A Jaeger, P Bibault and T Pottecher (CHU, Strasbourg); P Loirat and F Thaler (CH Foch, Suresnes); J Durand-Gasselin and I Granier (CHI, Toulon); M Génestal and O Anglès (CHU Purpan, Toulouse); C Virenque, K Samii and P Cougot (CHU Rangueil, Toulouse); H Georges (CH, Tourcoing); D Perrotin and V Gissot (CHU, Tours); A Gérard, C Meistelman, D Longrois and C Voltz (CHU Nancy-Brabois, Vandoeuvre-lès-Nancy); JP Bedos (CH, Versailles); and G Nitenberg and B Raynard (Institut Gustave Roussy, Villejuif).

## Appendix 2: the propensity score approach

Treatment comparisons can be conducted only if the populations being compared share common characteristics before they receive treatment. In randomized clinical trials, comparability is ensured by randomization of patients into different treatment groups. This process guarantees that observed as well as nonobserved characteristics are similar in the groups under study. In the present nonrandomized study, inclusion of a patient in one of the two groups was the result of a decision process guided by the drug availability and the choices of both the physician and the patient. There was no *a priori *reason to guarantee patient comparability in the two study phases. Recruitment bias was therefore expected from this type of two-phase design.

Steps have been taken to remove this bias. One of the most widely used criteria to identify recruitment biases is the balance of initial features between groups. This was done by standardizing their differences [19]. In effect, the difference between the means of a particular variable was weighted by its common standard deviation. If the observed difference between the two groups was significantly large compared with the variance of a particular variable, then the groups were deemed incomparable for that variable. The threshold of balance for any given variable was set at 10%. If a standardized difference for a variable was above 10%, then this meant that there was a recruitment bias on this variable.

Any recruitment bias must be controlled in order to allow appropriate comparison of costs. The propensity score is a well recognized method used to achieve this goal [20]. It indicates the probability that a subject with given characteristics will be exposed to treatment. It can reduce a large number of covariates into a single composite variable, which correctly summarizes all of the features observed. Its distribution provides a criterion with which to assess comparability between populations that are exposed or not exposed to treatment [21]. If two patients have similar scores, then it also means that they have similar initial characteristics.

The propensity score was estimated using a logistic regression model. The score was then used to construct a sample of comparable patients in the two phases using a matching process (that is, pairing a patient from the pre-license phase with a patient from the post-license phase who had a similar propensity score). The matching algorithm used was the SAS^© ^'match' macro [22]. This process is regarded as optimal because it matches patients from two different phases depending on their propensity score in order to minimize the total distance between the propensity score of matched patients (each distance representing the absolute value of the difference between the two propensity scores of the matched patient pair). The sample thus obtained is generally considered to be more balanced in terms of the observed features than the initial sample.
